# The Primacy of Beauty in Music, Visual Arts and Literature: Not Just a Replication Study in the Greek Language Exploring the Effects of Verbal Fluency, Age and Gender

**DOI:** 10.1177/00332941211026836

**Published:** 2021-06-19

**Authors:** Vaitsa Giannouli, Juliana Yordanova, Vasil Kolev

**Affiliations:** Bulgarian Academy of Sciences, Institute of Neurobiology, Sofia, Bulgaria

**Keywords:** Aesthetics, music, literature, visual arts, emotions, beauty, verbal fluency, working memory, aging, gender

## Abstract

Research on aesthetic descriptors of art in different languages is scarce. The aim of the present study was to elucidate the conceptual structure of aesthetic experiences of three forms of art (music, visual arts and literature) in the Greek language, which has not been explored so far. It was further aimed to study if biological and cognitive factors such as age and gender might produce differences in art appreciation. A total of 467 younger and older individuals from Greece were asked to generate verbal descriptors (adjectives) in free word-listing conditions in order to collect terms reflecting the aesthetics-related semantic field of art. The capacity of verbal memory was controlled by using a battery of neuropsychological tests. Analysis of generated adjectives’ frequency and salience revealed that ‘beautiful’ was the most prominent descriptor that was selected with a distinctive primacy for all three forms of arts. The primacy of ‘beautiful’ was significantly more pronounced for visual arts relative to music and literature. Although the aging-related decline of verbal capacity was similar for males and females, the primacy of ‘beautiful’ depended on age and gender by being more emphasized for young females than males, and for old males than females. Analysis of secondary descriptors and pairs of adjectives revealed that affective and hedonic experiences are essentially fixed in the semantic field of art reflection. It is concluded that although the concept of the aesthetics seems to be diversified and rich, a clear primacy of beauty is found for the Greek cultural environment and across different forms of art. The results also highlight the presence of complex influences of biological and cognitive factors on aesthetic art experiences.

## Introduction

As part of our everyday life, aesthetic experiences have been a long-standing topic of scientific interest (Fechner, 1876). However, no comprehensive scientific theory has been proposed to guide research on the nature of aesthetic reflections. Following different concepts and methodologies, the field has remained pluralistic for long times (Istók et al., 2009). Most recently elaborated links between empirical aesthetics and cognitive neuroscience provide new insights and suggest that aesthetic reflections emerge from neurocognitive mechanisms of perception, emotion, semantics, attention, and decision-making (Chatterjee & Vartanian, 2014, 2016; Pearce et al., 2016; Perlovsky, 2010; Skov & Nadal, 2020). Specifically, complex interactions between sensory-motor, emotion-valuation, and meaning-knowledge neural systems in the brain have been recognized as supporting aesthetic experiences (Chatterjee & Vartanian, 2014, 2016; Skov & Nadal, 2020). In order to explain phenomena central to aesthetics in general, and to perception of art, in particular (Perlovsky, 2010; Skov & Nadal, 2020), knowledge on how cognitive variables modulate aesthetic judgements needs to be further extended.

Within empirical aesthetics, one standard approach to measure aesthetic responses is to use stimuli (visual or auditory, neutral and/or emotionally charged, complex or simple stimuli, e.g., Giannouli, 2013a), and after their presentation, to examine how the individual feels about them (by answering to aesthetic judgment questions regarding participants’ mood, satisfaction, or other positive and negative statements) (Madsen et al., 1993). Within this procedure, aesthetic responses can be modulated or biased by the features of experimentally selected stimulation material.

Within another approach, emphasis is put on the conceptual structure of the aesthetics of objects, therefore responses do not necessarily follow stimulus presentation (Jacobsen et al., 2004). In such conditions, aesthetic terms are typically freely generated verbal descriptors, most frequently adjectives. Free word-association tasks have been used to examine the conceptual structure of aesthetic experiences and to draw inferences about the organization of the aesthetics-related semantic field (Deese, 1965; Jacobsen et al., 2004; Nelson et al., 2000). Establishing a semantic “map” of the field of aesthetics was first attempted in a systematic way by Jacobsen et al. (2004), who employed a free listing task to collect terms used for designating aesthetically relevant dimensions of objects. This original study revealed that “beauty” was a key center of the semantic field/structure of aesthetics since the adjective ‘beautiful’ was given by more than 90% of German-speaking participants (Jacobsen et al., 2004).

Notably, applying the same methodology has further revealed that “beauty” is a core concept also in aesthetics of art. In a study of Finnish-speaking participants, the adjective ‘beautiful’ proved to be the core item which was used to associate verbally the aesthetic value of music with appropriate adjectives (Istók et al., 2009). Likewise, when German-speaking participants were asked to list adjectives in order to label aesthetic dimensions of literature, the adjective ‘beautiful’ again ranked the highest frequency (Knoop et al., 2016). Thus, without prior presentation of a material to be reflected, the concept of “beauty” emerged as a core aesthetic reflection of art perception for different art modalities and for different languages. Such findings are important as providing links between general aesthetics of objects and aesthetics of art (Jacobsen et al., 2004; Skov & Nadal, 2020), the latter being associated with appraisal emotions at the highest levels of cognition and abstraction (Perlovsky, 2010). In addition, it is important to note that the information obtained by word associations reflects both lexical knowledge related to the specific linguistic system and conceptual knowledge acquired along life span (Nelson et al., 2004; Santos et al., 2011).

Although the importance of the adjective ‘beautiful’ is highlighted in both German and Finnish native language speakers (Istók et al., 2009; Jacobsen et al., 2004; Knoop et al., 2016), still there are no reports for languages that do not belong to the above linguistic group, as is the case with the modern Greek language. While both German and Finnish languages stem from the root of Proto-Germanic languages, the modern Greek language originates from a different and unique linguistic line of the Indo-European languages, being also the living language with the longest history (Malikouti-Drachman et al., 2018). In addition, aesthetic ideas of all west European countries are recognized as being based on ancient Greek ideals. With respect to such lexicon-specific and concept-grounding relevance of the Greek language, one major objective of the present study was to enable a cross-cultural examination of the semantic field of aesthetics of art by applying to Greek population the same protocol as in previous studies, in which German and Finish participants were investigated. For that aim, the established verbal method for generating spontaneous aesthetic descriptors (adjectives) was used (Jacobsen et al., 2004), according to which the set of words (or lexemes) related in meaning to aesthetics (semantic field) would be extracted through the lexical retrieval processes (Garrett, 1992). It was aimed to explore if the core concept of “beauty” would emerge as a major verbal descriptor of aesthetic reflections of art in Greek population, reflecting possible similarities or differences in the effects of linguistic/semantic or social factors on beauty appreciation.

Another objective was to extend this type of research by including different types of art – music, visual art, and literature - to explore if aesthetic verbal descriptors reflect generalized or art-specific aesthetics. The selection of three art conditions was based on the existence of similar observations in the Finnish language for the art of music (Istók et al., 2009) and in the German language for literature (Knoop et al., 2016), while visual arts were originally added here in order to compare the results from the two aforementioned arts to one additional artistic field that has remained unexplored. Recently, Che et al. (2018) have demonstrated that cross-cultural aesthetic preferences of objects are based on a common set of formal features including symmetry, complexity, proportion, contour, brightness, and contrast. However, Skov and Nadal (2020) have proposed to disentangle the aesthetic valuation of sensory objects from aesthetic art experiences. Since the aesthetic reflections of different arts are grounded on specific sensory modalities, comparing verbal aesthetic responses across arts would help highlight the issue about the role of objective sensory features in art aesthetics (Chatterjee & Vartanian, 2014; Perlovsky, 2010). Further, given the important proactive role that beauty plays in different aspects of everyday life (Konstan, 2014), elucidating the conceptual structure of aesthetics in different arts may support a number of future applications in relevant related fields (cultural products, marketing, culture industry, etc.).

A third objective of the present study was to provide further evidence for the influence of cognitive abilities on aesthetic reflections of art (Skov & Nadal, 2020) by exploring the effects of gender and age on aesthetic verbal descriptors. According to recent advances in neuroaesthetics, biological and cognitive factors essentially determine whether objects will be experienced as beautiful or not (Skov, 2019; Skov & Nadal, 2020). Aesthetic evaluations do depend on objective features composition (Che et al., 2018). However, the hedonic value of the aesthetic appraisal is increasingly recognized as a flexible category modulated by context, cognitive demands scaled by processing capacity and general knowledge, and emotional processing involving also reward responses (Skov & Nadal, 2020, 2021). Beauty judgments of art also are shown to engage executive processes supporting the formation of explicit evaluations in specific ways (Skov & Nadal, 2020).

In adulthood, gender differences have been found for each of the sensory systems that subserve perception and subsequent cognitive processes (Halpern, 2012). Males have most consistently demonstrated advantage in visual-spatial processing, especially mental rotation (Desrocher et al., 1995; Guerrieri et al., 2016; Vuoksimaa et al., 2010), while females have manifested superiority in language function (de Frias et al., 2006; Nicholson & Kimura, 1996; Willingham & Cole, 1997) and emotional processing (Abbruzzese et al., 2019; Olderbak et al., 2019; Sullivan et al., 2017; Thompson & Voyer, 2014; Tracy & Giummarra, 2017). Likewise, normal aging is associated with changes in cognition manifested by declines in cognitive control, attention, flexibility, inhibition, planning, verbal fluency, implicit decision-making, second-order and affective theory of mind (e.g., Calso et al., 2016; Cohen et al., 2019; Salthouse, 2010; Zanto & Gazzaley, 2019). Although emotion perception and emotional control do not exhibit an age-related decline (Zanto & Gazzaley, 2019), older adults are surprisingly more likely than young adults to report feeling positive (Gurera & Isaacowitz, 2019; Livingstone & Isaacowitz, 2019). Accumulating evidence about the effects of aging on lexical-semantic cognition further indicates that older adults’ semantic networks are less connected, less organized, and less efficient (e.g., Wulff et al., 2019; Krethlow et al., 2020). Together, these findings suggest that the complex alterations of cognitive faculties associated with gender and age may produce differences in beauty appreciation.

Yet, the way in which gender and aging might affect an individual's responses to art is an underexplored area (Pariser & Zimmerman, 1990). Preliminary studies have examined aesthetic evaluation only in adults and adolescents, showing higher and more positive evaluations in adults and a more elaborated way of aesthetic visual perception in females (Lin & IB, 2011). Other studies have supported the presence of gender differences not only in visual aesthetic perception, but also in music perception (Meyers-Levy & Zhu, 2010). A study of Finnish young college students has also shown that the choice of the adjective “beautiful” can be attributed to gender differences (i.e., male participants listed “beautiful” less frequently than females, Istók et al., 2009). In the present study, young student adults were included to provide a sample comparable to those used in similar studies (Istók et al., 2009; Jacobsen et al., 2004; Knoop et al., 2016), while older adults were added as a group that had not been investigated with respect to art aesthetics. We try to take into consideration both gender and age factors as it is not sufficiently well understood if and how aging-related alterations in cognitive abilities, life style, emotional intelligence etc. (Bakaev et al., 2007) may affect fundamental aesthetic reflections, and if gender may further modulate them. As an additional extension, the present study explored if the capacity of verbal memory and semantic realm might further affect aesthetic responses based on the production of verbal descriptors. The predictive value of three verbal memory tasks for aesthetic reflections was analyzed to explore if verbally produced aesthetic estimates might depend on or be confounded by individual verbal abilities.

Thus, the main questions addressed here were (1) is there a primacy of the aesthetic dimension of “beauty” in the adjectives that Greek people report for music, visual arts and literature, and (2) are biological (age, gender) and cognitive factors represented by verbal memory able to shape aesthetic adjectives production and therefore the semantic field/structure of aesthetics regarding these three forms of art?

## Method

### Participants

Two hundred and forty young (n = 240, mean age = 28.63, *SD* = 9.02, mean number of years of education = 14.39, *SD* = 1.64, 131 women) and two hundred and twenty seven older adults (n = 227, mean age = 72.23, *SD* = 6.57; mean number of years of education = 7.81, *SD* = 3.93, 137 women) from Greece coming from a larger pool of community-dwelling participants (Giannouli et al., 2019) participated in this study. All participants gave informed consent and were treated according to the Declaration of Helsinki. They were not explicitly informed about the aim of the experiment as they were told that some individual characteristics would be examined.

Young adults had no past or current psychiatric diagnosis or cognitive deficits, and reported standard and above academic achievements. None of the older adults has been clinically diagnosed with any cognitive deficit or neurodegenerative disorder. The cognitive status of older adults was assessed using the Mini Mental State Examination (MMSE) to exclude dementia and mild cognitive impairment, and the final sample included old participants who had a score larger than 27/30 points (Markwick et al., 2012). Some old adults were following medication related to heart problems/hypertension control or slight somatic complains. For both groups, exclusion criteria were a history of psychiatric or neurological problems, substance abuse-dependence, head injury or any other medical condition (including significant perceptual deficits such as visual and/or hearing impairments not corrected sufficiently by aids) that might affect neuropsychological performance. Non-native speakers of the Greek language also were excluded. All participants were non artists, and the current occupation of young adults was not related to music, visual arts and literature. Likewise, the previous professional occupation of retired old adults was not in the above artistic fields. The number of subjects included in the total sample of healthy adults satisfied criteria for a reliable statistical evaluation (Francis et al., 2010).

### Procedures and measurable parameters of aesthetic responses

#### Aesthetic responses

Participants filled out a demographic questionnaire addressing their age, gender, years and type of education, profession and occupation. Thereafter, they were asked to write down in five minutes as many descriptors-adjectives as possible regarding music, visual arts and literature (free listing task). The names of these three art forms were presented in counterbalanced order as we wished to control order effects in this repeated measures design. Thus, following previous protocols (Istók et al., 2009; Jacobsen et al., 2004), participants were given a blank one-page questionnaire with the following instruction: "Write down words that could be used to describe the aesthetics of music/literature/visual arts. Please use only adjectives". In order to distract the attention and memory of previous responses, participants completed the digit span forward task (Wechsler, 1955) and the phonological fluency test (Kosmidis et al., 2004) in the intermediate breaks between writing words/adjectives for each of the three forms of art (music-literature-visual arts, see next section for details).When any participant (n = 15) had questions about the exact meaning of the word ‘aesthetics’, an additional predefined explanation was presented: “Aesthetics refer to a branch of philosophy dealing with the appreciation of the nature of art and artistic creation”. Responses were given for music, literature and visual arts with varying order across participants and five-minute breaks between the conditions. A free listing task regarding three different arts was used to collect terms without imposing to the participants any answers. There was no specification of literature (e.g. specific mention to poem, novel, comedy, tragedy etc.), visual arts (e.g. painting, photography, sculpture, architecture etc.) or specific music genres, because a general response regarding all forms falling into these categories was emphasized. If some participants asked what exactly the visual arts included, they were told that the visual arts regarded visual objects such as pictures, faces, shapes, landscapes etc. As noted above, this protocol of measuring ‘language aesthetics’ was based on previously published research (Istók et al., 2009; Jacobsen et al., 2004), and was chosen to allow comparisons with existing cross-cultural relevant data. The following parameters were analyzed:

#### Total number of adjectives

Initially, the total number of adjectives generated by all participants was counted (including repetitions). As a second step, unreadable adjectives were excluded from analysis. In addition, adjectives that were reported by only one participant were removed accounting for the possibility that such words may not belong to the active vocabulary of the language (Sutrop, 2001). Only Greek adjectives or common foreign adjectives that are used in Greek were further analyzed (Babiniotis, 2012). After exclusions, the number of single adjectives used (without repetitions) was computed for all participants in the group.

It is to be mentioned the word ‘beautiful’ takes two forms in the Greek language (όμορφη/ωραία). According to the Greek language dictionaries of Triantafyllidis and Babiniotis, which are the best acknowledged dictionaries for modern Greek language (all versions in the last twenty years), there are no differences between the two synonyms in everyday use and in aesthetics. Therefore, the two forms were treated as one in the current analysis by creating a composite term. Nonetheless, results for the two separate adjectives also are presented (see Results).

Individual number of adjectives generated for aesthetic reflection of art was measured for each type of art after removing unreadable and repeated words.

Absolute frequency of occurrence was the total number of each adjective generated by all subjects in the group for each analyzed condition.

Relative frequency of occurrence was computed as the ratio between the total number of each respective adjective and the total number of all adjectives accepted for analysis generated by all subjects in the group for each analyzed condition. This index was introduced to reflect the distribution of the verbal descriptor in the lexical/semantic field of the condition-relevant population. It was expected that core descriptors would manifest a highest frequency, followed by peripheral descriptors and less relevant descriptors.

Mean list rank was computed as the group average position of each respective adjective generated for aesthetic reflection of art in each analyzed condition. The parameter was used to assess the salience of verbal descriptors.

Cognitive Salience Index (CSI). Following Sutrop (2001), a cognitive salience index (CSI) was calculated. CSI shows ‘the psychological salience in the list task combining the frequency and mean position of a term into one parameter. *CSI* is computed according to the equation

CSI = F/NmP,

where *F* is the frequency with which a term is named in the list task, *mP* is the mean position in which the term is named, and *N* is the number of subjects. Thus, if all subjects have named a term (*F* = *N*) and the mean position of that term is 1, then *CSI* is also 1 for that term (Sutrop, 2001).

#### Clustering

Co-occurrence of adjectives was computed as the normalized number of cases in the analyzed group (in %) where two adjectives (a pair) were present in the list of all generated adjectives. Clustering is generally found for both synonym and antonym words and is regarded as a relevant characteristic of the mental organization of the lexicon in all languages (Cacciari et al., 2015; Gjergo & Delija, 2014). Here, analysis of co-occurrence was conducted to characterize in more detail the organization of the lexical/semantic field associated with art-specific aesthetic reflections. In the Results, antonym and synonym pairing for “beautiful” is presented systematically as being the most frequent one showing both contrasting and synonym combinations.

### Verbal memory tests

During the intervals between the writing of aesthetic adjectives for the three art forms, participants performed the digit span forward task (Wechsler, 1955) and the verbal fluency test (Kosmidis et al., 2004). Data were collected through a paper-and-pencil way. No music, picture or text at this phase of the experiment was presented to the participants in order to avoid any bias. The examination material for the forward versions of the digit span were a series of improvised groups which consisted of one-digit numbers from 2 to 14 digits, which were read in a rate of one digit per second. Participants were required to repeat the sequences in the same order following the four listening conditions. A practice sequence of two digits was given for each task before the experiment started. Digit sequences were presented beginning with a length of two digits and two trials were presented at each increasing list length, but no stop criterion was used due the paper-and-pencil nature of the current testing. The versions of the word fluency test that were administered required the participants to produce in written form as many words as possible beginning with a specified letter from the Greek alphabet in a period of five minutes (phonemic fluency), and as many animals, fruits and objects (semantic fluency). These tests were employed because they impose strong demands on executive functioning, working memory and on (phonological and semantic) working memory (Rende et al., 2002; Rosen & Engle, 1997) including active search in long-term memory by means of phonemic-semantic cues, verbal response production, keeping track of the responses already given, and inhibition of irrelevant candidates. Half of the participants were examined first with the digit span forward task, and then with the phonological fluency test. The other half of the participants were given the same tests in a reverse order.

### Adjective valence rating

After the main sessions, an additional new sample of 28 participants, 10 men and 18 women (15 younger adults, mean age = 26.13, *SD* = 3.99, mean number of years of education = 14.13, *SD* = 1.68 and 13 older adults, mean age = 62.76, *SD* = 5.18, mean number of years of education = 14.76, *SD* = 1.42) completed a valence rating test, during which they rated the adjectives that the other participants produced (shown in Tables 2, 4, and 5) on a 7-point bipolar scale with anchors of -3 (negative) through 0 (neutral) to 3 (positive). This is a methodology that has been used in previous studies in other languages (Istók et al., 2009; Jacobsen et al., 2004) and it was used in order to examine whether the reported words in the Greek language have a positive or negative value for healthy young and old adults. This smaller group of naive participants followed the inclusion/exclusion criteria that were applied to the main larger sample of participants. For older adults again the MMSE was used for excluding the presence of dementia (score larger than 27/30 points).

### Statistical analyses

Measures from aesthetic responses in each art condition (number of adjectives produced for music, visual art and literature) and measures from the verbal memory tests (forward digit span test and the phonemic and semantic word fluency tests) were subjected to a two-factor analysis of variance (ANOVA) with between-subjects variables Age (young vs. old) and Sex (male vs. female). In additional regression analyses, it was tested if age (in years), years of education, forward digit span scores, phonological fluency scores and semantic fluency scores predicted the number of aesthetic adjectives for music, visual arts and literature was tested. A multiple stepwise regression was used to control for the possibility that the selected predictors might be inter-correlated. In a final analysis, the aesthetic responses parameters were compared between the three art conditions by including a within-subjects variable Art type (music vs. visual art vs. literature) in the ANOVA. For the valence ratings, descriptive statistics, such as means and standard deviations for each adjective, were computed. Between-group differences in the frequency of ‘beautiful’ appearance was tested using chi-square statistics.

## Results

### Demographic and neuropsychological data

Table 1 presents demographic data and results from neuropsychological testing of verbal memory. As expected, old adults had significantly less years of education than young adults. Yet, males and females did not differ with respect to age and years of education as indexed by non-significant Sex and Age x Sex effects. There was a main effect of Age on phonologic fluency scores, semantic fluency scores, and forward digit span scores, with young adults demonstrating better performance than old adults for all tests. However, main or interactive Sex effects were not significant.

**Table 1.** Demographic and neuropsychological assessment.

	Young male n = 109	Old male n = 90	Young female n = 131	Old female n = 137	Age *F*(1/466)	Sex *F*(1/466)	Age × Sex *F*(1/466)
Age (years)	29.7 ± 10.8	71.8 ± 6.4	27.8 ± 7.2	72.5 ± 6.7	3424.1*** *η_p_^2^*=0.88	0.6 *η_p_^2^*=0.001	3.24 *η_p_^2^*=0.007
Education (years)	14.4 ± 3.03	8.50 ± 2.94	14.4 ± 2.98	7.38 ± 2.93	539.0*** *η_p_^2^*=0.54	4.45* *η_p_^2^*=0.01	3.2 *η_p_^2^*=0.007
Forward digit span test	6.64 ± 1.67	6.25 ± 1.71	6.66 ± 1.72	6.08 ± 1.76	9.14** *η_p_^2^*=0.02	0.26 *η_p_^2^*=0.001	0.36 *η_p_^2^*=0.001
Phonological fluency test	25.1 ± 7.52	10.4 ± 7.50	24.9 ± 7.56	9.88 ± 7.49	439.2*** *η_p_^2^*=0.49	0.27 *η_p_^2^*=0.001	0.06 *η_p_^2^*=0.00
Semantic fluency test	26.5 ± 7.41	11.6 ± 7.40	25.9 ± 7.33	11.54 ± 7.37	444.6*** *η_p_^2^*=0.49	0.22 *η_p_^2^*=0.00	0.18 *η_p_^2^*=0.00

Note: For Age and Sex groups (the first four columns) mean values ± standard deviations are presented; n, number; *F*(*df*) is shown for the main factors and their interaction.

*p < 0.05; **p < 0.01; ***p < 0.001; *η_p_^2^*, partial eta squared.

### Verbal aesthetic descriptors

#### Music

For music, a total of 5749 Greek adjectives (including repetitions) were recorded from all participants. After exclusions, the number of single adjectives used for analysis (without repetitions) was 523.

**Figure 1.**
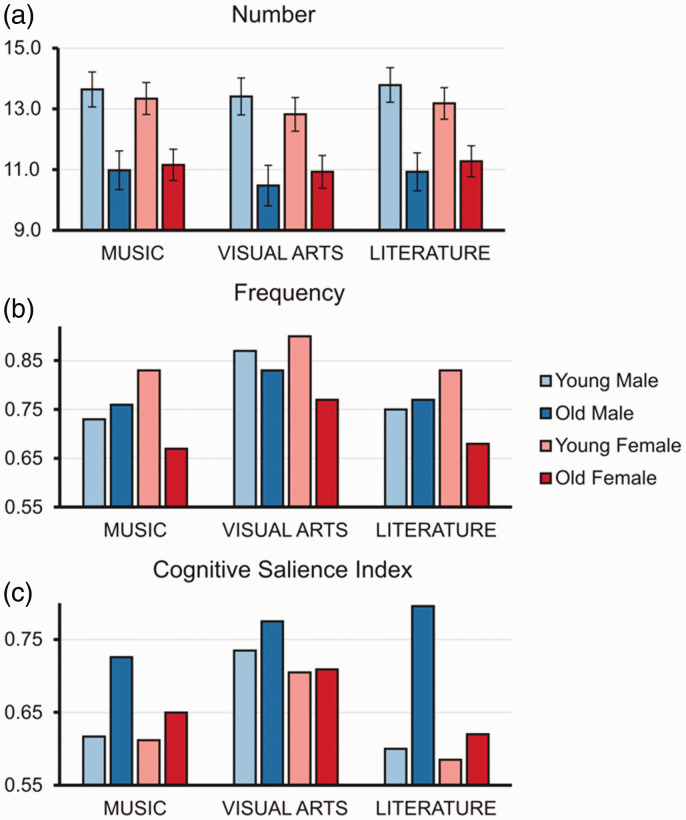
Group mean values of (a) number of adjectives for aesthetic art reflection, (b) relative frequency of ‘beautiful’, and (c) Cognitive Salience Index of ‘beautiful’ in four sub-groups of subjects – Young Male, Old Male, Young Female, Old Female for three types of art – MUSIC, VISUAL ARTS, and LITERATURE.

- Number. The mean individual number of adjectives generated for aesthetic reflection of music was 12.31 (*SD* = 6.13, minimum word production = 0, maximum word production = 34). Figure 1(a) demonstrates that the number of reported adjectives was significantly higher in young (13.47, *SD* = 5.88) relative to old adults (11.08, *SD* = 6.16), Age (*F*(1, 463) = 18.32, *p* = 0.000, *η_p_^2^* = 0.04). No Sex effects were yielded.

**Table 2.** Relative frequencies (percentage), absolute frequencies, mean list rank, and cognitive salience index (CSI) of the most used single adjectives for music in the Greek language.

Adjectives	Relative frequency of occurrence (%)	Absolute frequencies	Mean list rank	CSI
beautiful (όμορφη/ωραία)	74.0%	345	1.01	0.731
relaxing (χαλαρωτική)	64.6%	302	5.44	0.118
depressive (καταθλιπτική)	63.8%	298	4.84	0.131
fast (γρήγορη)	56.7%	265	5.95	0.095
slow (αργή)	56.0%	262	7.19	0.007
good (καλή)	52.9%	247	2.66	0.198
bad (κακή)	50.6%	236	9.50	0.053
calming (γαλήνια)	50.4%	235	10.10	0.049
hard (δύσκολη)	49.2%	230	22.63	0.021
ugly (άσχημη)	48.0%	224	7.70	0.062
pleasant (ευχάριστη)	47.4%	221	10.31	0.045
touching (συγκινητική)	45.6%	213	2.31	0.197
peaceful (ειρηνική)	44.5%	208	10.07	0.044
atmospheric (ατμοσφαιρική)	43.8%	205	12.58	0.034
original (αυθεντική)	37.8%	177	21.69	0.017
sad (λυπηρή)	37.4%	175	14.10	0.026
dull (βαρετή)	37.4%	175	16.26	0.023
warm (ζεστή)	35.2%	164	21.48	0.016
impressive (εντυπωσιακή)	33.1%	155	16.10	0.020
loud (δυνατή)	32.4%	151	15.10	0.021
bright (λαμπερή)	28.7%	134	23.69	0.012
rhythmic (ρυθμική)	27.4%	128	16.03	0.017
unforgettable (αξέχαστη)	25.3%	118	18.58	0.013

Note: The English translation of the adjective ‘beautiful’ in the analyses included two Greek words (όμορφη/ωραία), which are synonyms. They were analyzed as a single composite variable, because of their identical meaning in the Greek language.
- Frequency. As depicted in Table 2, the most frequently named term (frequency rate = 74%) that was used for aesthetic reflection of music was ‘beautiful’ (represented by the two words that are used interchangeably in Greek: όμορφη/ωραία). Figure 1(b) demonstrates that Age (*χ*^2^ = 2.6, *p* = 0.1) and Sex (*χ*^2^ = 0.02, *p* = 0.1) did not affect the frequency of “beautiful” appearance. However, young females selected the term “beautiful” significantly more frequently than old females (*χ*^2^ = 6.4, *p* = 0.01). With “beautiful” being the core aesthetic descriptor, the next words with high frequency were “relaxing” (χαλαρωτική) and “depressive” (καταθλιπτική) and represented the relevant peripheral aesthetic descriptors of music in Greek (64.6% and 63.8%, respectively).
- CSI. As shown in Figure 1(c) and Table 2, the Cognitive Salience Index revealed a highest salience for “beautiful” confirming the term as a core descriptor. Notably, the aesthetic reflection “beautiful” had a stronger salience in old relative to young adults, which was especially emphasized in old males – Figure 1(c). Although the frequency of “beautiful” was reduced in old relative to young females, the bias towards the term increased with age also in females.
- Clustering. Co-occurrence of adjectives was found for pairs of specific words, and more specifically for opposites on bipolar dimensions, such as good-bad (50.7%) and slow-fast (41.3%). For the core descriptor ‘beautiful’ there were highly frequent combinations, the most frequent being those of contrasting categories: beautiful-relaxing (49.1%), beautiful-depressive (48.8%), and beautiful-ugly (48%) – see Figure 2 for detailed presentation.

**Figure 2.**
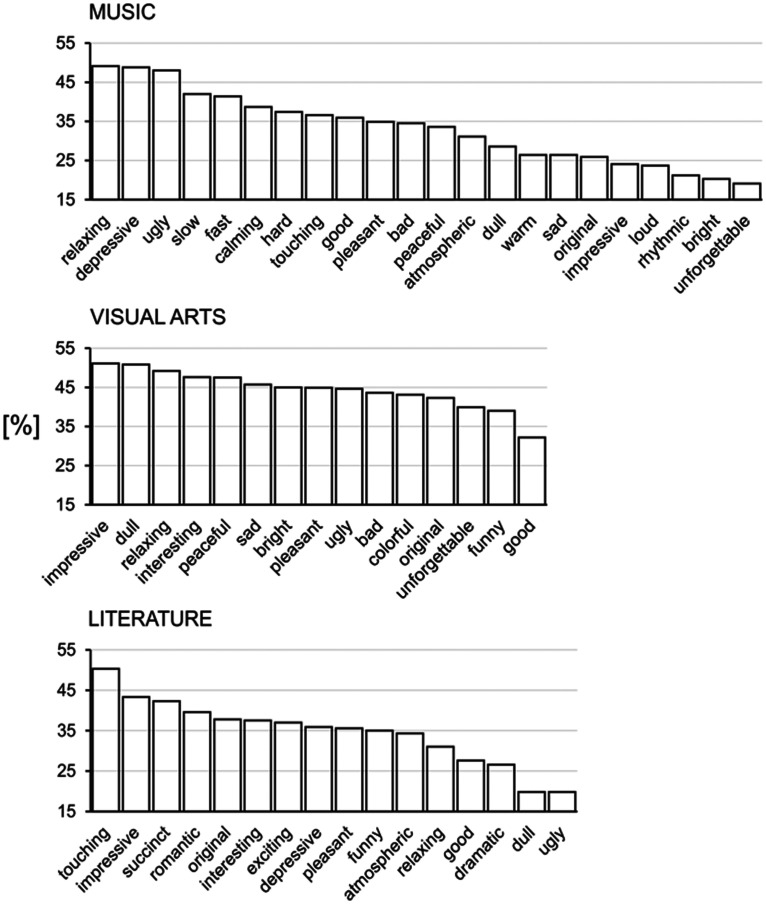
Relative frequency (in %) of appearance of verbal descriptors in combination with the core descriptor ‘beautiful’ for MUSIC, VISUAL ARTS, and LITERATURE.

#### Visual arts

For visual arts, a total of 5582 Greek adjectives were recorded from all participants including the identical adjectives. Following the procedure for excluding unreadable and subject-unique adjectives, the final number of single adjectives used for visual arts aesthetics was 572.

- Number. The mean individual number of adjectives related to visual arts aesthetics was 11.95 (*SD* = 6.43, minimum word production = 0, maximum word production = 34). There was a statistically significant main effect of Age (*F*(1, 463) = 16.49, *p* = 0.000, *η_p_^2^* = 0.03) due to a higher number in young (13.09, *SD* = 6.34) relative to old adults (10.74, *SD* = 6.31). No Sex effects were yielded.
- Frequency. As depicted in Table 3, again the most frequently named adjective (a core descriptor) was ‘beautiful’ (83.7%). Figure 1(b) demonstrates that old age was associated with a significant decrease in the frequency of ‘beautiful’ appearance (Age, *χ*^2^ = 6.4, *p* = 0.01), which was mainly due to a significantly lower frequency only in old females (Age effect in females, *χ*^2^ = 7.2, *p* = 0.007). According to frequency of appearance, the relevant peripheral aesthetic descriptors of visual arts in Greek were dull (βαρετή) and impressive (εντυπωσιακή) (61.0% and 60.0%, respectively) – Table 3.

**Table 3. table3-00332941211026836:** Relative frequencies (percentage), absolute frequencies, mean list rank, and cognitive salience index (CSI) of the most used single adjectives for visual arts in the Greek language.

Adjectives	Relative frequency of occurrence (%)	Absolute frequencies	Mean list rank	CSI
beautiful (όμορφη/ωραία)	83.7%	391	1.06	0.789
dull (βαρετή)	61.0%	285	8.95	0.068
impressive (εντυπωσιακή)	60.0%	280	4.12	0.145
relaxing (χαλαρωτική)	58.2%	272	14.51	0.040
interesting (ενδιαφέρουσα)	54.7%	255	4.97	0.109
bright (λαμπερή)	54.4%	254	9.68	0.056
pleasant (ευχάριστη)	54.4%	254	2.15	0.252
peaceful (ειρηνική)	54.2%	253	15.56	0.034
sad (λυπηρή)	51.6%	241	13.48	0.038
bad (κακή)	51.4%	240	11.64	0.044
colorful (πολύχρωμη)	50.7%	237	7.83	0.064
original (αυθεντική)	50.5%	236	5.96	0.084
funny (αστεία)	47.9%	224	10.71	0.044
unforgettable (αξέχαστη)	47.5%	222	6.93	0.068
ugly (άσχημη)	46.9%	219	12.93	0.036
good (καλή)	37.4%	175	3.11	0.120

Note: The English translation of the adjective ‘beautiful’ in the analyses included two Greek words (όμορφη/ωραία), which are synonyms. They were analyzed as a single composite variable, because of their identical meaning in the Greek language.
- CSI. As shown in Figure 1(c) and Table 3, “beautiful” manifested the highest salience as indicated by the CSI (0.789). Again, the salience of the aesthetic reflection “beautiful” was stronger in old males as compared to the other age and sex sub-groups. In females, the preference for “beautiful” did not change with age.
- Clustering. Co-occurrence of adjectives was found for pairs of specific words, the most frequent being beautiful-impressive (51.1%), beautiful-dull (50.8%), beautiful-relaxing (49.2%) – Figure 2.

#### Literature

In total, 5758 Greek adjectives including identical adjectives were recorded from all participants for aesthetic reflection of literature. Thirty-two adjectives were excluded from the analysis, because they were unreadable. After all exclusions, the number of single adjectives used for literature aesthetics was 412.

(A) Number. On average, the whole group of participants produced 12.33 (*SD* = 6.04, minimum word production = 2, maximum word production = 34) adjectives. The number of generated adjectives was larger in young (mean = 13.45, *SD* = 5.3) than old adults (mean = 10.74, *SD* = 6.31; Age, *F*(1, 463) = 18.25, *p* = 0.000, *η_p_^2^* = 0.04), whereas no Sex differences were found.

(B) Frequency. Table 4 demonstrates that the most frequently generated adjective to reflect aesthetics of literature (a core descriptor) was again ‘beautiful’ (75.1%). As depicted in Figure 1(b), for literature too, old participants tended to produce the adjective ‘beautiful’ less frequently (Age, *χ*^2^ = 3.2, *p* = 0.07). Yet, the frequency of the core adjective was significantly lower in old as compared to young females (Age effect in females, *χ*^2^ = 6.6, *p* = 0.01). As peripheral aesthetic descriptors of literature in Greek the adjectives “touching” (συγκινητική) and “impressive” (εντυπωσιακή) were extracted (65.0% and 57.1%, respectively) – Table 4.

**Table 4.** Relative frequencies (percentage), absolute frequencies, mean list rank, and cognitive salience index (CSI) of the most used single adjectives for literature in the Greek language.

Adjectives	Relative frequency of occurrence (%)	Absolute frequencies	Mean list rank	CSI
beautiful (όμορφη/ωραία)	75.1%	351	1.07	0.724
touching (συγκινητική)	65.0%	304	2.66	0.244
impressive (εντυπωσιακή)	57.1%	267	7.13	0.080
succinct (περιληπτική)	57.1%	267	17.38	0.032
dramatic (δραματική)	55.8%	261	3.19	0.175
interesting (ενδιαφέρουσα)	55.1%	257	4.36	0.126
depressive (καταθλιπτική)	52.5%	245	6.33	0.082
romantic (ρομαντική)	51.4%	240	5.00	0.102
bad (κακή)	50.6%	236	15.08	0.033
original (αυθεντική)	49.2%	230	7.90	0.062
pleasant (ευχάριστη)	47.5%	222	8.85	0.053
atmospheric (ατμοσφαιρική)	46.5%	217	18.52	0.025
exciting (συναρπαστική)	46.0%	215	9.80	0.046
funny (αστεία)	44.9%	210	12.20	0.036
peaceful (ειρηνική)	44.5%	208	20.31	0.021
relaxing (χαλαρωτική)	44.3%	207	12.70	0.034
sad (λυπηρή)	37.4%	175	17.52	0.021
good (καλή)	36.4%	170	10.63	0.034
bright (λαμπερή)	28.7%	134	21.20	0.013
dull (βαρετή)	25.7%	120	13.43	0.019
unforgettable (αξέχαστη)	25.3%	118	16.55	0.015
ugly (άσχημη)	20.9%	98	21.37	0.009

Note: The English translation of the adjective ‘beautiful’ in the analyses included two Greek words (όμορφη/ωραία), which are synonyms. They were analyzed as a single composite variable, because of their identical meaning in the Greek language.

(C) CSI. Figure 1(c) and Table 3 show that similar to other arts, “beautiful” manifested the highest salience (0.724). Notably, as found for music, the salience of the aesthetic descriptor “beautiful” was remarkably enhanced in old males as compared to all other age and sex sub-groups, whereas the CSI in old females was not substantially changed.

(D) Clustering. As demonstrated in Figure 2, a more frequent co-occurrence of adjectives was found for the pairs comprising ‘beautiful’ - beautiful-touching (50.3%), beautiful-impressive (43.3%), and beautiful-succinct (42.3%).

#### Comparison between arts

One-way repeated-measures analysis of variance (ANOVA) was used to test the effect of art type (music vs. visual art vs. literature) on the number of adjectives generated as art descriptors. There was a significant effect of type of art, due to a smaller number of adjectives generated for visual art (11.95, *SD* = 6.4) as compared to music (12.3, *SD* = 6.13) and literature (12.3, *SD* = 6.04), *F*(2/465) = 17.656, *p* < 0.001, *η_p_^2^* = 0.071).

As described before, for each of the three arts ‘beautiful’ was the most frequently generated adjective. Yet, the effect of art type on the frequency of ‘beautiful’ was significant (*χ^2^* = 76.2, *p* < 0.001) because ‘beautiful’ was generated significantly more frequently for visual arts relative to music (*χ^2^* = 70.4, *p* < 0.001) and literature (*χ^2^* = 9.68, *p* = 0.002) – Figure 1(b). Likewise, the CSI of ‘beautiful’ was highest for visual arts relative to music and literature – Figure 1(c).

### Control analyses

#### ‘Beautiful’: ‘Όμορφη’ or ‘Ωραία’?

When the two adjectives in Greek which are translated in English as ‘beautiful’, (‘όμορφη’ and ‘ωραία’) were treated as separate words, relative frequencies (percentage) for music were 71% (N = 331; mean list rank = 1; cognitive salience index = 0.708) for ‘ωραία’, and only 3% (N = 14; mean list rank = 24.2; cognitive salience index = 0.001) for ‘όμορφη’. Accordingly, for visual arts frequencies were 79,7% (N = 372; mean list rank = 1; cognitive salience index = 0.796) for ‘ωραία’ and only 4% (N = 19; mean list rank = 18; cognitive salience index =0.055) for ‘όμορφη’ . Finally, for literature frequencies were 73,1% (N = 342; mean list rank = 1; cognitive salience index = 0.732) for ‘ωραία’ and only 2% (N = 9; mean list rank = 25.4; cognitive salience index = 0.039) for ‘όμορφη’. In all cases where individuals reported the adjective ‘όμορφη’, also the adjective ‘ωραία’ was also reported at the very beginning of the list. Thus, the two terms were listed together for all forms of art, but in different positions in the lists.

### Verbal memory and aesthetic descriptors

Multiple stepwise regression analyses with phonological fluency, semantic fluency, forward digit span, age, and years of education as independent variables and the number of aesthetic adjectives (for music, visual arts and literature separately) as a dependent variable, revealed that only performance in the phonological fluency test predicted in a statistically significant way the number of adjectives for the aesthetics of music (*F*(1/466) = 55.7, *p* < 0.001), visual arts (*F*(1/466) = 55.5, *p* < 0.001), and literature (*F*(1/466) = 56.42, *p* < 0.001) - Table 5. No other predictors (age, years of education, performance in the semantic fluency, or the digit forward tasks) were yielded.

**Table 5.** Contribution of verbal fluency to aesthetic adjectives for the arts.

	Predictor	*β*	*Beta*	*t*	*p*	*R* ^2^_adj_
Visual aesthetic number of adjectives	Phonological verbal fluency test	0.20	0.33	7.45	0.001	0.11
Literature aesthetic number of adjectives	Phonological verbal fluency test	0.19	0.33	7.51	0.001	0.11
Music aesthetic number of adjectives	Phonological verbal fluency test	0.19	0.33	7.47	0.001	0.11

Note: *β, Beta, t, p, R*^2^_adj_, regression analysis parameters; *R*^2^_adj,_ adjusted *R^2^*.

### Valence ratings

Valence ratings obtained from the 28 additional participants showed that the majority of adjectives in the Greek language (n = 26) produced by the large sample (Tables 2 [Table table3-00332941211026836]to 4) have a positive value, thus rendering the arts descriptors not only affective, but also positively affective in valence. This is obvious by the number of adjectives (n = 18) that were characterized as positively charged, in contrast to the negatively charged adjectives (n = 8, hard, slow, bad, loud, dramatic, sad, ugly, and depressive, see mean scores of Likert responses for the adjectives in Table 6).

**Table 6.** Valence ratings for reported adjectives.

Adjectives	Minimum	Maximum	Mean	SD
Beautiful (όμορφη)	2.00	3.00	2.60	0.49
Nice (ωραία)	2.00	3.00	2.67	0.47
Touching	−2.00	3.00	1.53	1.13
Pleasant	1.00	3.00	2.32	0.61
Atmospheric	1.00	3.00	1.57	0.74
Warm	1.00	3.00	2.35	0.73
Hard	−3.00	1.00	−1.21	1.13
Slow	−3.00	2.00	−0.32	1.12
Fast	−2.00	2.00	0.39	0.87
Original	1.00	3.00	1.96	0.74
Bad	−3.00	0.00	−1.39	1.03
Good	1.00	3.00	2.35	0.67
Calming	0.00	2.00	1.60	0.56
Peaceful	1.00	3.00	2.07	0.71
Impressive	1.00	3.00	2.17	0.61
Loud	−3.00	1.00	−1.10	1.34
Rhythmic	0.00	2.00	1.25	0.79
Exciting	1.00	3.00	2.25	0.64
Succinct	−1.00	3.00	1.50	0.96
Interesting	0.00	3.00	1.96	0.74
Funny	1.00	3.00	2.21	0.62
Romantic	−1.00	3.00	1.89	1.03
Dramatic	−3.00	−1.00	−2.03	0.74
Sad	−3.00	1.00	−1.64	0.98
Ugly	−3.00	−1.00	−2.25	0.64
Depressive	−3.00	−1.00	−2.14	0.75

Note: SD, standard deviation.

## Discussion

The present study explored the organization of the semantic field and the conceptual structure of aesthetics in several art dimensions (music, visual arts, and literature) in Greek population, considering also the effects of biological and cognitive factors (age and gender).

### The core concept of ‘beauty’ in art aesthetics in the Greek language

For all three forms of art, the parameters frequency and cognitive salience index were markedly enhanced for the descriptor ‘beautiful’, thus extracting the primacy of the ‘beauty’ concept for art reflection and strongly differentiating it from other descriptors. These observations reveal for the first time the importance of ‘beauty’ in aesthetic responses of Greek population, emphasizing the fundamental cross-cultural significance of the ‘beauty’ concept in the organization of the semantic reflection of art aesthetics.

In the Greek lexicon, two terms ‘όμορφη’ and ‘ωραία’ are used as synonyms corresponding to the English descriptor ‘beautiful’. The high frequency of cases when the two terms are listed together for all forms of art emphasizes the close connection between the two adjectives, but at the same time, the different and distant positions of these two words in the lists reveals that the adjective ‘ωραία’ is more important for aesthetics of art in the Greek language.

The adjective ‘beautiful’ (combining the two synonyms in Greek) had a rather high prevalence (74% for music, 83% for visual arts, 75% for literature), as all other terms were markedly less frequently and less saliently produced (Tables 2 [Table table3-00332941211026836]to 4). Yet, this adjective production was lower in frequency compared to the German language (91.6% for the aesthetics of objects, not specified according to the artistic field; Jacobsen et al., 2004) and was higher in frequency compared to the Finnish language (66% for music; Istók et al., 2009). It is not plausible to relate these differences to the size or wealth of aesthetic verbal production, since the average number of adjectives per young person in Greece was 13.12, it was slightly lower in Finland (12.4; Istók et al., 2009), and in a similar sample of young adults from Germany it was much lower (9.4; Jacobsen et al., 2004). These differences may be due not only to some cross-cultural factors, but as in the case of Jacobsen et al. (2004), the non-specification of the type of aesthetic objects may have crystalized the prominence of the ‘beauty’ concept. Furthermore, currently observed aging effects demonstrate that ‘beautiful’ was the most conspicuous descriptor in old adults despite the aging-dependent reduction of the number of generated aesthetic terms. Hence, the primacy of ‘beauty’ in art aesthetics may not be directly associated with the richness of explicitly extracted descriptors. Instead, the dominance of ‘beauty’ appears as a function of the structure of the semantic field of art (Istók et al., 2009).

In this respect, it is interesting that in the present study, there were no terms with frequency of occurrence less than 20% for all three forms of art, pointing to a homogeneity of the semantic field of art aesthetics in the Greek language. This is in contrast to the results in German (Jacobsen et al., 2004) and Finnish languages (Istók et al., 2009), as well as to findings according to which the usage of words for aesthetic impressions follows an exponential distribution (Augustin et al., 2012). These cross-cultural similarities in the prevalence of ‘beauty’ and differences in the overall organization of the semantic field of art aesthetics can be interpreted in light of the overall debate regarding aesthetics and the possible differences even across western cultures in connecting sensory experience to cognition and to social structure, which has yet to be developed in a more comprehensive way (Coleman, 2011).

### The nature of peripheral descriptors and clustering

Aesthetic responses in Greek hardly represent a homogeneous concept despite the prevalence of the adjective ‘beautiful’ (in a manner that seems to be perseverative, Giannouli, 2013b). Indeed, a number of adjectives were generated with relatively higher frequency thus appearing as peripheral descriptors. First, and most important, these terms were related to the hedonic value of art reflection and did not directly refer to the physical features of each specific art modality (Tables 2 [Table table3-00332941211026836]to 4). The observation that the majority of produced adjectives were descriptors of affective characteristics points to the emotion-relatedness of peripheral descriptors and reveals that emotional impressions are essentially fixed in the semantic domain of art reflection. These results support the leading role of the hedonic evaluation in the aesthetic experience of art (Skov & Nadal, 2020) and also support models of aesthetic experience that emphasize the main involvement of affective states (Leder et al., 2004; Reber et al., 2004; Silvia, 2005). Second, these peripheral descriptors were not identical for the three types of art (music, visual arts, and literature), suggesting that the emotional states accompanying the aesthetic valuation process are specific for each art. Third, the peripheral adjectives produced most frequently (forming clusters at frequency of appearance at around 60%) were not fully overlapping with the most salient additional adjectives. This result implies that the penetration of a descriptor in the explicit report may be biased by different factors such as the frequency of usage of the word in everyday life in general depending on education, occupation, or environment, the strength of associations between the aesthetic experience and a given term in the semantic network, etc. (Coleman, 2011).

In the present study, the organization of the semantic field of art aesthetics also was implicated by the frequency of descriptors co-occurrence (clustering). Clustering is generally found for words that share similar properties in the verbal fluency test for the American as well as the Greek language (Kosmidis et al., 2004). However, the occurrence of antonym pairs (containing opposite words) is more robust in the lexico-semantic relations, relevant to the mental organization of the lexicon subserving the everyday human communication in all languages (Cacciari et al., 2015; Gjergo & Delija, 2014). Antonymy is regarded as the most robust of the semantic relations in all languages, relevant to both the mental organization of the lexicon and the organization of coherent discourse (Fellbaum, 1998; Jones, 2002; Murphy, 2003; Paradis et al., 2009; van de Weijer et al., 2014; Willners, 2001). In this line, the clusters of semantically contrasting-opposites for aesthetic adjectives may show the importance of a hypothetical semantic network, where antonyms of adjectives appear stronger than synonyms in some cases. This may be apparent in the process of recalling relevant antonym or even synonym adjectives from long-term memory which come from this hypothetical cognitive dipole, something that subsequently shapes future aesthetic responses in the direction of directly expressing the dipole (by saying an antonym) or in the direction of indirectly expressing the dipole (by saying synonyms that are based on this hypothetical contrast). Therefore, both antonymy and synonymy are expected in verbal semantic tasks.

In aesthetics of music, the ‘beautiful-ugly’ dimension has been previously found to represent a primary bipolar concept, appearing also as a cross-cultural language semantic similarity (Jacobsen et al., 2004; Lorand, 1994). According to the present results from the Greek language, synonym but not antonym combinations with ‘beautiful’ appeared with highest frequency (beautiful-relaxing, beautiful-impressive, and beautiful-touching for music, visual arts and literature, respectively). Furthermore, with respect to antonym pairing, the dipole adjective pair ‘beautiful-ugly’ was indeed present in the reports, but other dipoles were more prevalent for each of the arts – music (beautiful-depressive), visual arts (beautiful – dull), and literature (beautiful – depressive) - Figure 2. These findings of both synonym and antonym pairs generated by Greek participants may be explained by the fact that, as discussed above, mainly the affective perception of art experience was reflected in the selected adjectives, which produced the most frequent combinations (Menninghaus et al., 2020). In contrast, the ‘beautiful-ugly’ dipole may be a higher-order abstraction (Perlovsky, 2010) reflecting essentially a philosophical dimension of art conceptualization. Hence, despite the cross-cultural similarities in the primacy of ‘beautiful’ in the semantic organization of the lexicons, peripheral descriptors and clustering in the present study helped to reveal that the primary selection of ‘beautiful’ in Greek population was perhaps more closely linked to a hedonic connotation of this descriptor.

### Effects of art type—Music, visual arts, literature

The results of the present study provide original evidence for the specific structure of lexical/semantic field of aesthetic experience of visual arts among other types of art explored here (music and literature). First, the number of adjectives generated for visual arts (mean 11.9) was significantly smaller than the number of descriptors generated for music (mean 12.3) and literature (mean 12.3). This result implies that the aesthetic experience of visual arts is least explicit and verbalized, remaining probably mostly at the implicit sub-conscious level. In addition, both the frequency and salience of ‘beautiful’ were significantly higher for visual arts relative to music and literature. This original result reveals for the first time that the primacy of ‘beauty’ (at least in the Greek language) is especially dominant for visual arts. As these effects did not stem from age or gender, they might have originated from the strongest links between the aesthetic term ‘beautiful’ and appreciations of visual objects (in the visual modality). Considering in parallel current findings from peripheral descriptors and clustering, this observation is also important by implying that the primacy of ‘beauty’ in aesthetic experiences of art has a complex origin and is supported by at least three different connotations - reference to a harmonic structure of physical features (Che et al., 2018; Reber et al., 2004), reference to a hedonic appreciation and a positive emotional state (Reber et al., 2004; Silvia, 2005; Skov & Nadal, 2020), and reference to an affirmative conceptual abstraction (Perlovsky, 2010). While visual arts integrate all sources producing a highest salience, the relatively lower salience of ‘beautiful’ in aesthetic responses to literature (Table 4; Knoop et al., 2016) may reflect a limited contribution of the ‘visual/physical modality’ dimension (Reber et al., 2004).

### Effects of age and sex

Another original finding of the present study was that gender efficiently modified the semantic field of aesthetic art experience. Notably, gender effects only emerged in older age. Current analysis of the frequency and salience of the descriptor ‘beautiful’ revealed that the primacy of ‘beauty’ in terms of both frequency and salience was strikingly enhanced in older males for all types of art. In old females, in contrast, the frequency of appearance of the adjective decreased. While the higher mean frequency for young females suggests that the concept of beauty is less important or less central for males (Istók et al., 2009), possibly due to superiority of females in emotional (Abbruzzese et al., 2019; Olderbak et al., 2019; Sullivan et al., 2017; Tracy & Giummarra, 2017) and verbal processing (de Frias et al., 2006; Nicholson & Kimura, 1996; Willingham & Cole, 1997), the inverse pattern in the older group is a completely new finding. These gender-dependent reversal was observed on the background of reduced capacity for word generation in old participants, which was essentially predicted by phonologic verbal fluency (Table 5). Yet, the aging-related suppression of verbal memory was similar for males and females (Table 1), thus refuting the possibility that verbal processing capacity was responsible for the observed differences in the old group. Rather, the age-dependent reversal of gender effects may be due to a greater abstraction or better visualization strategies in older males (Guerrieri et al., 2016; Reber et al., 2004; Vuoksimaa et al., 2010) as a result of both sex-dependent differences in visual-spatial skills (Guerrieri et al., 2016) and in the higher level of education in old males (Table 1). However, future research is needed to clarify these effects and aesthetic responses in other languages and cultural environments, and explore the underlying neural and psychological mechanisms (Nieminen et al., 2011).

In summary, the present study demonstrates that although the concept of the aesthetics still seems to be diversified and rich, a clear primacy of beauty is found for the Greek cultural environment and across different forms of art. The results provide original evidence for a) the dominating primacy of beauty in visual arts as compared to music and literature; b) the leading role of affective and hedonic experiences in the semantic field of art reflection; and c) the presence of complex influences of biological and cognitive factors (age and gender) on aesthetic art experiences. Further neurocognitive studies are needed to shed light on the neurophysiologic grounds of these effects.

## References

[bibr1-00332941211026836] Abbruzzese L. Magnani N. Robertson I. Mancuso M. (2019). Age and gender differences in emotion recognition. Frontiers in Psychology, 10, 2371. 10.3389/fpsyg.2019.02371 31708832 PMC6819430

[bibr2-00332941211026836] Augustin M. D. Wagemans J. Carbon C. C. (2012). All is beautiful? Generality vs. specificity of word usage in visual aesthetics. Acta Psychologica, 139(1), 187–201. 10.1016/j.actpsy.2011.10.004 22123506

[bibr3-00332941211026836] Babiniotis G. (2012). Dictionary of modern Greek language. Center Lexicology.

[bibr4-00332941211026836] Bakaev M. Lee K. H. Cheng H. I. (2007, October). The aesthetic and emotional preferences of the elderly and the design factors for e-business web sites. In *The Eighth Pan-Pacific Conference on Occupational Ergonomics (PPCOE 2007)*.

[bibr5-00332941211026836] Cacciari C. Pesciarelli F. Gamberoni T. Ferlazzo F. (2015). Is black always the opposite of white? The comprehension of antonyms in schizophrenia and in healthy participants. In *Ceur Workshop Proceedings* (Vol. 1347, pp. 166–171). 10.3390/bs5010093 PMC4384065 25760930

[bibr6-00332941211026836] Calso C. Besnard J. Allain P. (2016). Normal aging of frontal lobe functions. Geriatrie et Psychologie Neuropsychiatrie du Vieillissement, 14(1), 77–85. (in French). 10.1684/pnv.2016.0586 27005339

[bibr7-00332941211026836] Chatterjee A. Vartanian O. (2014). Neuroaesthetics. Trends in Cognitive Sciences, 18(7), 370–375. 10.1016/j.tics.2014.03.003 24768244

[bibr8-00332941211026836] Chatterjee A. Vartanian O. (2016). Neuroscience of aesthetics. Annals of the New York Academy of Sciences, 1369(1), 172–194. 10.1111/nyas.13035 27037898

[bibr9-00332941211026836] Che J. Sun X. Gallardo V. Nadal M. (2018). Cross-cultural empirical aesthetics. Progress in Brain Research, 237, 77–103. 10.1016/bs.pbr.2018.03.002 29779752

[bibr10-00332941211026836] Coates J. (2015). Women, men and language: A sociolinguistic account of gender differences in language. Routledge.

[bibr11-00332941211026836] Cohen R. A. Marsiske M. M. Smith G. E. (2019). Neuropsychology of aging. Handbook of Clinical Neurology, 167, 149–180. 10.1016/B978-0-12-804766-8.00010-8 31753131

[bibr12-00332941211026836] Coleman E. B. (2011). Aesthetics as a cross-cultural concept. Literature & Aesthetics, 15(1), 57–78.

[bibr13-00332941211026836] de Frias C. Nilsson L. G. Herlitz A. (2006). Sex differences in cognition are stable over a 10-year period in adulthood and old age. Neuropsychology, Development, and Cognition. Section B, Aging, Neuropsychology and Cognition, 13(3–4), 574–587. 10.1080/13825580600678418 16887790

[bibr14-00332941211026836] Deese J. (1965). The structure of associations in language and thought. Johns Hopkins Press.

[bibr15-00332941211026836] Desrocher M. Smith M. Taylor M. (1995). Stimulus and sex differences in performance of mental rotation: Evidence from event-related potentials. Brain and Cognition, 28(1), 14–38. 10.1006/brcg.1995.1031 7546666

[bibr16-00332941211026836] Fechner G. T. (1876). *Vorschule der aesthetik* [Aesthetics preschool] (Vol. 1). Breitkopf & Härtel.

[bibr17-00332941211026836] Fellbaum C. (Ed.). (1998). WordNet: An electronic lexical database. MIT Press.

[bibr18-00332941211026836] Foos P. W. Clark M. C. (2011). Adult age and gender differences in perceptions of facial attractiveness: Beauty is in the eye of the older beholder. The Journal of Genetic Psychology, 172(2), 162–175. 10.1080/00221325.2010.526154 21675545

[bibr19-00332941211026836] Francis J. J. Johnston M. Robertson C. Glidewell L. Entwistle V. Eccles M. P. Grimshaw J. M. (2010). What is an adequate sample size? Operationalising data saturation for theory-based interview studies. Psychology and Health, 25, 1229–1245. 10.1080/08870440903194015 20204937

[bibr20-00332941211026836] Garrett M. F. (1992). Lexical retrieval processes: Semantic field effects. In A. Lehrer, & E. F. Kittay (Eds.), *Frames, fields and contrasts: New essays in semantic and lexical organization* (pp. 377–395). Routledge.

[bibr21-00332941211026836] Giannouli V. (2013a). Visual symmetry perception. Encephalos, 50, 31–42.

[bibr22-00332941211026836] Giannouli V. (2013b). Number perseveration in healthy subjects: Does prolonged stimulus exposure influence performance on a serial addition task? Advances in Cognitive Psychology, 9(1), 15–19. 10.2478/v10053-008-0127-8 23717347 PMC3664543

[bibr23-00332941211026836] Giannouli V. Kolev V. Yordanova J. (2019). Is there a specific Vivaldi effect on verbal memory functions? Evidence from listening to music in younger and older adults. Psychology of Music, 47(3), 325–341. 10.1177/0305735618757901

[bibr24-00332941211026836] Gjergo E. S. Delija S. (2014). The role and function of the antonyms in language. Mediterranean Journal of Social Sciences, 5(16), 703–705.

[bibr25-00332941211026836] Guerrieri G. M. Wakim P. G. Keenan P. A. Schenkel L. A. Berlin K. Gibson C. J. Rubinow D. R. Schmidt P. J. (2016). Sex differences in visuospatial abilities persist during induced hypogonadism. Neuropsychologia, 81, 219–229. 10.1016/j.neuropsychologia.2015.12.021 26719236 PMC4768303

[bibr26-00332941211026836] Gurera J. W. Isaacowitz D. M. (2019). Emotion regulation and emotion perception in aging: A perspective on age-related differences and similarities. Progress in Brain Research, 247, 329–351. 10.1016/bs.pbr.2019.02.007 31196440

[bibr27-00332941211026836] Halpern D. (2012). Sex differences in cognitive abilities (4th ed.). Psychology Press, Taylor & Frances Group.

[bibr28-00332941211026836] Istók E. Brattico E. Jacobsen T. Krohn K. Müller M. Tervaniemi M. (2009). Aesthetic responses to music: A questionnaire study. Musicae Scientiae, 13(2), 183–206.

[bibr29-00332941211026836] Jacobsen T. Buchta K. Köhler M. Schröger E. (2004). The primacy of beauty in judging the aesthetics of objects. Psychological Reports, 94(3c), 1253–1260. 10.2466/pr0.94.3c.1253-1260 15362400

[bibr30-00332941211026836] Jones S. (2002). Antonymy: A corpus-based perspective. Routledge.

[bibr31-00332941211026836] Knoop C. A. Wagner V. Jacobsen T. Menninghaus W. (2016). Mapping the aesthetic space of literature “from below”. Poetics, 56, 35–49.

[bibr32-00332941211026836] Konstan D. (2014). *Beauty: The fortunes of an ancient Greek idea*. Oxford University Press.

[bibr33-00332941211026836] Kosmidis M. H. Vlahou C. H. Panagiotaki P. Kiosseoglou G. (2004). The verbal fluency task in the Greek population: Normative data, and clustering and switching strategies. Journal of the International Neuropsychological Society, 10(2), 164–172. 10.1017/S1355617704102014 15012836

[bibr34-00332941211026836] Krethlow G. Fargier R. Laganaro M. (2020). Age-specific effects of lexical-semantic networks on word production. Cognitive Science, 44(11), e12915. 10.1111/cogs.12915 33164246 PMC7685158

[bibr35-00332941211026836] Leder H. Belke B. Oeberst A. Augustin D. (2004). A model of aesthetic appreciation and aesthetic judgments. British Journal of Psychology, 95(4), 489–508. 10.1348/0007126042369811 15527534

[bibr36-00332941211026836] Lin Y. C. , & IB. (2011). Generation and gender differences in aesthetic responses to popular illustrations. Visual Arts Research, 37(1), 30–41.

[bibr37-00332941211026836] Livingstone K. M. Isaacowitz D. M. (2019). Age similarities and differences in spontaneous use of emotion regulation tactics across five laboratory tasks. Journal of Experimental Psychology: General, 148(11), 1972–1992. 10.1037/xge0000556 30714783 PMC6679823

[bibr38-00332941211026836] Lorand R. (1994). Beauty and its opposites. The Journal of Aesthetics and Art Criticism, 52(4), 399–406.

[bibr39-00332941211026836] Madsen C. K. Brittin R. V. Capperella-Sheldon D. A. (1993). An empirical method for measuring the aesthetic experience to music. Journal of Research in Music Education, 41(1), 57–69.

[bibr40-00332941211026836] Malikouti-Drachman A. Newton B. E. Ruijgh C. J. Lejeune M. (2018). *Greek language*. Encyclopedia Britannica.

[bibr41-00332941211026836] Markwick A. Zamboni G. de Jager C. A. (2012). Profiles of cognitive subtest impairment in the Montreal Cognitive Assessment (MoCA) in a research cohort with normal Mini-Mental state examination (MMSE) scores. Journal of Clinical and Experimental Neuropsychology, 34(7), 750–757. 10.1080/13803395.2012.672966 22468719

[bibr42-00332941211026836] Menninghaus W. Schindler I. Wagner V. Wassiliwizky E. Hanich J. Jacobsen T. Koelsch S. (2020). Aesthetic emotions are a key factor in aesthetic evaluation: Reply to Skov and Nadal (2020). Psychological Reviews, 127(4), 650–654. 10.1037/rev0000213 32584122

[bibr43-00332941211026836] Meyers-Levy J. Zhu R. J. (2010). Gender differences in the meanings consumers infer from music and other aesthetic stimuli. Journal of Consumer Psychology, 20(4), 495–507.

[bibr44-00332941211026836] Murphy L. M. (2003). Semantic relations and the lexicon: Antonymy, synonymy and other paradigms. Cambridge University Press.

[bibr45-00332941211026836] Nelson D. L. McEvoy C. L. Dennis S. (2000). What is free association and what does it measure? Memory & Cognition, 28(6), 887–899. 11105515 10.3758/bf03209337

[bibr46-00332941211026836] Nelson D. L. McEvoy C. L. Schreiber T. A. (2004). The University of South Florida free association, rhyme, and word fragment norms. Behavior Research Methods, Instruments & Computers, 36(3), 402–407. 10.3758/BF03195588 15641430

[bibr47-00332941211026836] Nicholson K. Kimura D. (1996). Sex differences for speech and manual skill. Perceptual and Motor Skills, 82(1), 3–13. 10.2466/pms.1996.82.1.3 8668494

[bibr48-00332941211026836] Nieminen S. Istók E. Brattico E. Tervaniemi M. Huotilainen M. (2011). The development of aesthetic responses to music and their underlying neural and psychological mechanisms. Cortex, 47(9), 1138–1146. 10.1016/j.cortex.2011.05.008 21665202

[bibr49-00332941211026836] Olderbak S. Wilhelm O. Hildebrandt A. Quoidbach J. (2019). Sex differences in facial emotion perception ability across the lifespan. Cognition & Emotion, 33(3), 579–588. 10.1080/02699931.2018.1454403 29564958

[bibr50-00332941211026836] Paradis C. Willners C. Jones S. (2009). Good and bad opposites: Using textual and psycholinguistic techniques to measure antonym canonicity. The Mental Lexicon, 4(3), 380–429.

[bibr51-00332941211026836] Pariser D. Zimmerman E. (1990). Editorial: Gender issues in art education. Studies in Art Education, 32(1), 3–5.

[bibr52-00332941211026836] Pearce M. T. Zaidel D. W. Vartanian O. Skov M. Leder H. Chatterjee A. Nadal M. (2016). Neuroaesthetics: The cognitive neuroscience of aesthetic experience. Perspectives on Psychological Science, 11(2), 265–279. 10.1177/1745691615621274 26993278

[bibr53-00332941211026836] Perlovsky L. I. (2010). Intersections of mathematical, cognitive, and aesthetic theories of mind. Psychology of Aesthetics, Creativity, and the Arts, 4(1), 11–17. 10.1037/a0018147

[bibr54-00332941211026836] Ramachandran V. S. Hirstein W. (1999). The science of art: A neurological theory of aesthetic experience. Journal of Consciousness Studies, 6(6–7), 15–51.

[bibr55-00332941211026836] Reber R. Schwarz N. Winkielman P. (2004). Processing fluency and aesthetic pleasure: Is beauty in the perceiver's processing experience? Personality and Social Psychology Review, 8(4), 364–382. 10.1207/s15327957pspr0804_3 15582859

[bibr56-00332941211026836] Rende B. Ramsberger G. Miyake A. (2002). Commonalities and differences in the working memory components underlying letter and category fluency tasks: A dual-task investigation. Neuropsychology, 16(3), 309–321. 10.1037//0894-4105.16.3.309 12146678

[bibr57-00332941211026836] Rosen V. M. Engle R. W. (1997). The role of working memory capacity in retrieval. Journal of Experimental Psychology: General, 126(3), 211–227. 9281831 10.1037//0096-3445.126.3.211

[bibr58-00332941211026836] Salthouse T. A. (2010). Selective review of cognitive aging. Journal of the International Neuropsychological Society, 16(5), 754–760. 10.1017/S1355617710000706 20673381 PMC3637655

[bibr59-00332941211026836] Santos A. Chaigneau S. E. Simmons W. K. Barsalou W. (2011). Property generation reflects word association and situated simulation. Language and Cognition, 3(1), 83–119. 10.1515/langcog.2011.004

[bibr60-00332941211026836] Silvia P. J. (2005). Emotional responses to art: From collation and arousal to cognition and emotion. Review of General Psychology, 9(4), 342–357.

[bibr61-00332941211026836] Skov M. (2019). The neurobiology of sensory valuation. In M. Nadal & O. Vartanian (Eds.), *The Oxford handbook of empirical aesthetics* (pp. 1–40). Oxford University Press. 10.1093/oxfordhb/9780198824350.013.7

[bibr62-00332941211026836] Skov M. Nadal M. (2020). A farewell to art: Aesthetics as a topic in psychology and neuroscience. Perspectives on Psychological Science, 15(3), 630–642. 10.1177/1745691619897963 32027577

[bibr63-00332941211026836] Skov M. Nadal M. (2021). The nature of beauty: Behavior, cognition, and neurobiology. Annals of the New York Academy of Sciences, 1488(1), 44–55. 10.1111/nyas.14524 33147651

[bibr64-00332941211026836] Sullivan S. Campbell A. Hutton S. Ruffman T. (2017). What’s good for the goose is not good for the gander: Age and gender differences in scanning emotion faces. The Journals of Gerontology: Series B, 72(3), 441–447. 10.1093/geronb/gbv033 25969472

[bibr65-00332941211026836] Sutrop U. (2001). List task and a cognitive salience index. Field Methods, 13(3), 263–276.

[bibr66-00332941211026836] Thompson A. E. Voyer D. (2014). Sex differences in the ability to recognise non-verbal displays of emotion: A meta-analysis. Cognition & Emotion, 28(7), 1164–1195. 10.1080/02699931.2013.875889 24400860

[bibr67-00332941211026836] Tracy L. M. Giummarra M. J. (2017). Sex differences in empathy for pain: What is the role of autonomic regulation? Psychophysiology, 54(10), 1549–1558. 10.1111/psyp.12895 28555773

[bibr68-00332941211026836] van de Weijer J. Paradis C. Willners C. Lindgren M. (2014). Antonym canonicity: Temporal and contextual manipulations. Brain and Language, 128(1), 1–8. 10.1016/j.bandl.2013.10.011 24269667

[bibr69-00332941211026836] Vuoksimaa E. Kaprio J. Kremen W. S. Hokkanen L. Viken R. J. Tuulio-Henriksson A. Rose R. J. (2010). Having a male co-twin masculinizes mental rotation performance in females. Psychological Sciences, 21(8), 1069–1071. 10.1177/0956797610376075 PMC4438761 20581340

[bibr70-00332941211026836] Wechsler D. (1955). Wechsler adult intelligence scale. Psychological Corporation.

[bibr71-00332941211026836] Willingham W. Cole N. (1997). Gender and fair assessment. Lawrence Erlbaum Assoc.

[bibr72-00332941211026836] Willners C. (2001). *Antonyms in context: A corpus-based semantic analysis of Swedish descriptive adjectives* (Vol. 40). Lund University.

[bibr73-00332941211026836] Wulff D. U. De Deyne S. Jones M. N. Mata R. , & Aging Lexicon Consortium. (2019). New perspectives on the aging lexicon. Trends in Cognitive Sciences, 23(8), 686–698. 10.1016/j.tics.2019.05.003 31288976

[bibr74-00332941211026836] Xia X. (2013). Gender differences in using language. Theory and Practice in Language Studies, 3(8), 1485–1489.

[bibr75-00332941211026836] Zanto T. P. Gazzaley A. (2019). Aging of the frontal lobe. Handbook of Clinical Neurology, 163, 369–389. 10.1016/B978-0-12-804281-6.00020-3 31590742

